# Challenges and Perspectives of Neurological Disorders: Series II

**DOI:** 10.3390/brainsci15080887

**Published:** 2025-08-20

**Authors:** Dina Nur Anggraini Ningrum, Woon-Man Kung

**Affiliations:** 1 Public Health Department, Faculty of Medicine, Universitas Negeri Semarang, Semarang 50229, Indonesia; 2 Division of Neurosurgery, Department of Surgery, Taipei Tzu Chi Hospital, Buddhist Tzu Chi Medical Foundation, New Taipei City 23142, Taiwan; 3 Department of Exercise and Health Promotion, College of Kinesiology and Health, Chinese Culture University, Taipei 11114, Taiwan

Neurological disorders (NDs) has become the foremost public health threat across the globe in recent years, resulting in upsetting and devastating outcomes. Therefore, there is an urgent need to improve the effects of NDs on individuals. Stroke is a common ND that communities should be aware of. Investigators have illustrated the high incidence of stroke burden today. The predicted worldwide expense of cerebrovascular accidents is over USD 890 billion, which represents 0.66% of the global gross domestic product [1].

With increasing life spans in modern populations compounding environmental risk exposures, the prevalence of age-related neurodegenerative illnesses is quietly rising. The development of NDs can be caused by neuronal dysfunctions during the pathogenic inflammatory process [2]. Furthermore, it is critical to comprehend how free radical production, neuroinflammation, neurotoxicity, and disease progression interact in order to clarify illness mechanisms and develop treatment plans that target and mitigate the advancement of disease [3]. This means that novel studies that protect neurons from oxidative stress and lessen neuroinflammation are desperately required. The recent literature has discussed the functions of tau pathology in NDs such as Alzheimer’s disease (AD). Tau take part in neuronal dysfunction, inflammation, oxidative damage, and mitochondrial impairment that are part of the etiology of these disorders [4]. The global scientific community is now advancing interdisciplinary collaboration and technological convergence at an extraordinary pace in several domains, aiming to redefine the future of NDs through precision treatment and visionary wellness interventions. Traditional diagnostic and therapeutic models are inadequate for addressing the expanding complexity of disease profiles. Recent advances in machine learning (ML) have led to promising outcomes in precise recognition of Parkinson’s disease (PD) through deep learning (DL) [5]. ML and DL models have even been used to diagnose depression through the examination of electroencephalograph (EEG) data [6]. Apart from traditional management strategies, herbal and non-invasive interventions, including yoga, meditation, traditional pressure point therapy, massage, and other external manipulations may bring about a synergistic solution to the troublesome context of neurological care, thereby creating novel therapeutic avenues [7]. 

By outlining the significant demand for the development of new approaches, modeling tools, and software, which aim to improve our comprehension of biological or clinical data, our goal was to facilitate ongoing dialog on research computation and inform collective efforts to address challenges and perspectives on NDs. The combination of multiple sophisticated techniques and robust and carefully interpreted and validated multidimensional datasets from population-based cohorts will result in the improved explanatory accuracy of NDs. The current Special Issue (SI) is an extension of a previous series published in 2023 [8]. We received a total of 13 submissions for the current publication. After a vigorous review process, we decided to publish eight articles and reject five of them. The aim of the papers included in this SI is to summarize new key findings, explore challenges and successes, share personal anecdotes, and reflect on implications for future research.

Rehabilitation exercise is essential for maximal functional recovery of stroke patients [9]. A non-randomized trial conducted by Giarmatzis and colleagues applied a 12-week moderate-to-high intensity muscle strengthening program using Pilates equipment to individuals who had survived a stroke. They concluded that the implemented intervention was able to enhance both force production and control of gait, particularly in affected limbs, resulting in better ambulation ability (Contribution 1).

Similarly, Doskas et al. published an in-depth narrative review discussing the neurological and neurocognitive sequelae of coronavirus disease 2019 (COVID-19), with particular emphasis on their connection to AD. It is interesting that AD shares several similarities with the cognitive impairments that result from long-COVID-19. Severe acute respiratory syndrome coronavirus type 2 can influence the central nervous system via two mechanisms, either directly, through viral entry, or indirectly, through systemic inflammation and immune dysregulation (Contribution 2).

A large cross-sectional cohort study conducted by Zeng et al. found that the apolipoprotein E (APOE) genotype, specifically the APOEε4 allele, is associated with cognitive deficits. It plays a potential role in the advancement and severity of cognitive disorders, including AD, with cognitive function declining more significantly with age in ε4 carriers, especially among women (Contribution 3).

Most recently, a brain age prediction model trained using tau brain images was proposed by Wang and his team to accurately generate brain age gap predictions for cognitively impaired victims in the AD continuum, which could be a valuable biomarker for detecting and monitoring AD (Contribution 4).

The authors from Ling’s laboratory investigated a radiomics-guided DL model using DenseNet in order to significantly improve the identification and medical diagnosis of Parkinsonian syndromes, including idiopathic PD, multiple system atrophy, and progressive supranuclear palsy. Their work highlights the potential to extract quantitative features using DL from medical images obtained through 18F-fluorodeoxyglucose positron emission tomography scanning, with the aim of achieving high diagnostic accuracy (Contribution 5).

Meanwhile, the paper by Suzuki et al. presents another groundbreaking finding: a ML model is able to accurately detect depression using EEG data obtained from easy and readily available consumer-grade EEG devices. They reported and demonstrated promising accurate depression detection by quantifying various EEG indices and employing feature selection using a light gradient boosting machine model (Contribution 6).

The survey by Xu et al. concluded that a traditional Chinese herbal dietary formula significantly improved both cognitive and physical functions in elderly participants with mild cognitive impairments. In contrast to the placebo group, the herbal formula group showed distinct improvements in their physical function and ability to perform daily life activities, as well as enhanced cognitive scores, leading to obvious clinical application value (Contribution 7).

Tsiakiri et al. enrolled patients in a cross-sectional study to analyze what influences the ability of individuals with neurocognitive disorders to perform instrumental activities of daily living (IADL), which are crucial daily tasks like managing finances or preparing meals. The results emphasized the critical role of IADL assessment in developing effective support strategies from a clinical viewpoint (Contribution 8).

Thus, we are excited to showcase our upcoming SI: “Challenges and Perspectives of Neurological Disorders: Series II”. This series continues our commitment to present the latest perspectives, challenges, and innovative solutions in the field of NDs.
